# Cytogenetic Analyses in Ewes with Congenital Abnormalities of the Genital Apparatus

**DOI:** 10.3390/ani9100776

**Published:** 2019-10-10

**Authors:** Sara Albarella, Emanuele D’Anza, Giacomo Galdiero, Luigi Esposito, Davide De Biase, Orlando Paciello, Francesca Ciotola, Vincenzo Peretti

**Affiliations:** Department of Veterinary Medicine and Animal Production, University of Naples Federico II, via Delpino 1, 80137 Naples, Italy; emanuele.danza@unina.it (E.D.); galdiero.giacomo@hotmail.it (G.G.); luigespo@unina.it (L.E.); davide.debiase@unina.it (D.D.B.); orlando.paciello@unina.it (O.P.); francesca.ciotola@unina.it (F.C.); vincenzo.peretti@unina.it (V.P.)

**Keywords:** ewe, disorder of sex development (DSD), XX/XY cell chimaerism

## Abstract

**Simple Summary:**

The disorders of sex development (DSDs) are congenital conditions characterized by inconsistency among chromosomal, gonadal, and anatomical sex development. Reproduction and prolificacy are two main parameters in the sheep industry; thus, DSDs in sheep are very detrimental. Interestingly, no DSDs-associated gene mutations have been reported in sheep so far, probably due to the fact that affected animals are not detected and studied. With the aim to deepen the knowledge about DSDs in sheep and improve diagnostic tools, screening of a sheep farm aimed to detect and study DSDs in ovine species has been started, and the actual findings relative to the first two sheep flocks analysed are reported here. In our opinion, this study demonstrates that, despite the low number of studies on ovine DSDs, if compared with other species, this problem is actually present and needs more attention.

**Abstract:**

The Disorders of Sex Development (DSDs) are congenital conditions characterized by inconsistency among chromosomal, gonadal, and anatomical sex development. The aim of this research is to report the clinical and cytogenetic findings of four DSD cases and 13 couples of heterosexual twins in sheep. To this purpose, C- and R-banding techniques were used, and the analyses of the *SRY* (Sex Determining Region Y) and *AMEL* (Amelogenin) genes were carried out. Moreover, morphopathological analyses were performed in one case. The four DSD sheep cases were registered as females at birth, and for none of them it was possible to establish whether the subjects were born from heterosexual multiple births. Three of the four cases were diagnosed as XX/XY blood lymphocyte chimaeras, while the fourth case was diagnosed as a 54, XY *SRY*-positive DSD sheep. None of the heterosexual twins showed XX/XY blood chimaerism. This finding suggests that the blood chimaeric cases detected could also be due to a zygote/embryo fusion. Moreover, no gene variants involved in sheep DSD are known, the identification of which would be very useful for the sheep industry.

## 1. Introduction

One of the main causes of subfertility or sterility in livestock is Disorders of Sex Development (DSDs), congenital conditions in which there is an inconsistency among chromosomal, gonadal, and anatomical sex development [1,2].

The study of livestock affected by these disorders allows the discovery of new causative mutations and the development of new diagnostic tools to identify and eliminate them from breeding populations. Moreover, it provides the opportunity to improve the understanding of mammalian sexual development and differentiation.

Sheep DSD cases reported in the literature are mainly linked to XX/XY lymphocyte cell chimaerism caused by blood mixing through vascular anastomosis developed between heterosexual twins during multiple pregnancies (freemartin syndrome) [3]. This finding is of great interest, because freemartin syndrome in sheep is not as frequent in the case of multiple births with lambs of different sex. In fact, different studies on the incidence of freemartin cases have shown variability between 1.6 and 8%, according to the analysed breed [4,5,6]. This condition could be explained by the effect of a gene influencing the formation of placental anastomosis among twins. Some studies based on pedigree analysis showed a familial tendency toward vascular anastomoses at the early stage in gestation, supposing the existence of a single dominant gene influencing this trait [4,7,8]. The knowledge of this percentage in those populations and breeds that are in selection for litter size is of great interest. This is because the number of multiple births with heterosexual lambs is increasing, and the chance of selecting freemartin lambs as future ewes will depend upon this percentage. On the other hand, the prolificacy rate is the most relevant economic trait in the sheep industry [9]; thus, its improvement is often among the selection aims of sheep breeders.

Other sheep cases of DSDs have been associated with a variety of chromosomal abnormalities involving sex chromosomes: XX/XY full body cell chimaerism [10,11]; XX/X0 cell chimaerism [12]; 55,XXY karyotype [13]; and a pure X chromosome monosomy [14]. Interestingly, only one case of XY sex reversal syndrome, *SRY*-positive and with no alteration in *SRY* sequence, has been reported [15]. The affected ewe showed normal external genitalia, and the only visible alteration was a short vulva opening length.

Thus, no gene mutations associated with DSDs have been reported in sheep up to now. This is probably due to the management of this species because of which some DSD individuals with an almost normal phenotype are not detected and thus are not reported (above all when they are considered male at birth) and some DSD ewes showing sterility are directly culled without submitting them to any type of analysis. In fact, the most studied DSD in sheep is freemartin syndrome due to the fact that the affected animals have a history of sterility associated to a birth with new-borns of different sex. Thus, DSD animals may represent a cause of hidden economic losses in the sheep industry, but being rarely detected and reported, they are also poorly known and studied, and no diagnostic tools for early detection of DSDs are available. The future prospects for the use of reproductive biotechnologies in this species make the development of such tools indispensable. In fact, such tools will prevent the use of carriers of mutations responsible for DSDs in reproduction, a practice that would result in the rapid spread of unfavourable genes in the ovine, with extremely negative consequences for the species.

With the aim to deepen the knowledge about DSD in sheep and to improve diagnostic tools, two sheep flocks have been studied, and four cases of DSD in sheep have been detected and then phenotypically and genetically characterized. Preliminary results about this research are displayed in this paper.

## 2. Material and Methods

### 2.1. Ethical Statement

All procedures used in this research were approved by the Ethical Animal Care and Use Committee of the University of Naples Federico II.

### 2.2. Cases

Cases 1, 2 and 4 belonged to a farm with 1250 sheep. The breeder reports that there was only 1 DSD case/year.

Case 3 was detected on a farm with 50 sheep. According to the breeder, there was only 1 DSD case/8 years.

Both farms are located in the province of Viterbo.

All the animals were registered as females at birth, and it was not possible to establish whether any of the subjects were born from heterosexual multiple births.

Because of the high frequency of DSDs in the first farm, we analysed all the heterosexual twins (13 couples) born in the next reproductive season. All the twins were evaluated with C- and R-banding and with *SRY* and *AMELX/Y* analyses to verify if they were chimaeras or not.

### 2.3. Morphological Analyses

Only for Case 1, it was possible to carry out morphopathologic analysis. The animal was slaughtered in strict accordance with the European slaughter regulations (CE n° 1099/2009), and the internal and external genital organs were observed for the detection of pathologic lesions or abnormalities.

Samples from testis and reproductive tissue were collected and preserved in 10% neutral buffered formalin (code no. 05-01007Q, Bio-Optica, Milan, Italy), dehydrated and embedded in paraffin (code no. 06-7920, Bio-Optica, Milan, Italy). Tissue sections were stained with haematoxylin and eosin (HE) for morphological analysis.

### 2.4. Cytogenetic Analyses

Blood lymphocytes were cultured in RPMI medium (BE12-702F, Lonza, Basel, Switzerland) with Concanavalin A (code no. C2010, Sigma-Aldrich, St. Luis, AZ, USA) and 10% of FBS, Australian origin, (code no. 10099141, GIBCO, New York, NK, USA) for about 72 h at 37.5 °C. Two types of cultures, with and without 5-BrdU (code no. B5002, Sigma-Aldrich, St. Luis, AZ, USA), were set up. A total of 20 µg/mL of 5-BrdU (code no. B5002, Sigma-Aldrich, St. Luis, AZ, USA) and H33258 (40 ug/mL) (code no. B1155, Sigma-Aldrich, St. Luis, AZ, USA) were added to the latter 5 h before harvesting. Colcemid (code no. L0040, Microtech srl, Naples, Italy) was added 1 h before harvesting to all cultures. A hypotonic treatment with 0.075 M KCl (code no. 471177, Carlo Erba, Rodano MI, Italy) and three fixations with Carnoy’s fixative were performed. Cell suspensions were used to prepare slides that were allowed to dry and then stained for C- and R-banding. A total of 200 and 10 metaphases were examined from slides with Acridine Orange staining, treated for C- and R-banding techniques, respectively (CBA and RBA). Karyotypes were arranged according to Cribiu et al. [16].

### 2.5. Molecular Analyses

DNA was extracted from whole blood with Wizard^®^ Genomic DNA purification kit (Promega) and tested by qualitative PCR using primers specific for *SRY* and *AMELY/X*. Table 1 shows primers, PCR conditions and the length of the fragments.

## 3. Results

### 3.1. Clinical Findings

**Figure 1:**
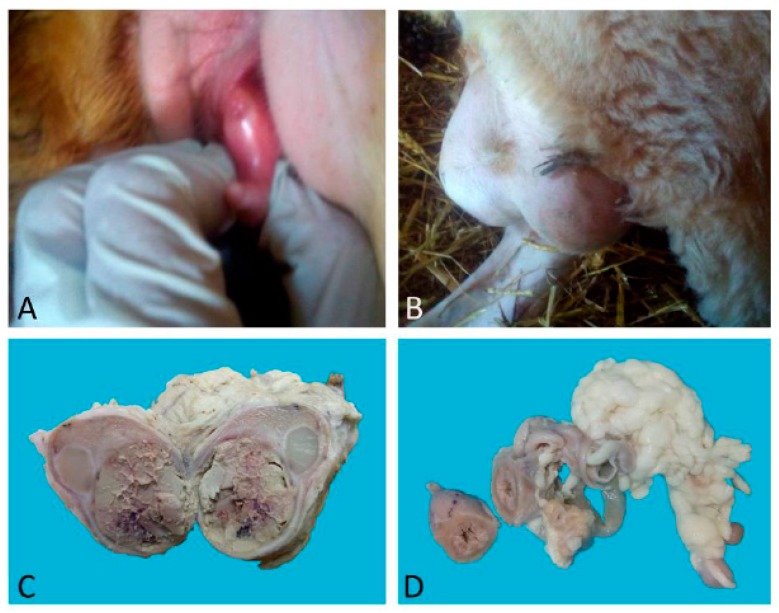
Case 1. (**A**) Posterior view in which a protruding penis-like clitoris is evident; (**B**) inguinal region in which two evident masses are visible; (**C**,**D**) transversal sections of one of the testes and of the tubular structure found during necroscopy.

Case 1 was a nulliparous 14-month-old half-breed ewe with male phenotypic traits: the presence of horns and a big heavy head. Sexual behaviour was that of a ram, while the vulva had a ventral margin facing upwards with a penis-like protruding clitoris (Figure 1A). The vagina was blind-ending. In the inguinal region, two testes-like structures were detected (Figure 1B) that sonographically showed the echogenicity of liver-like parenchyma with cavitations and hyperechoic point areas. In the middle of the mass, a hyperechoic sept was detected.

**Figure 2:**
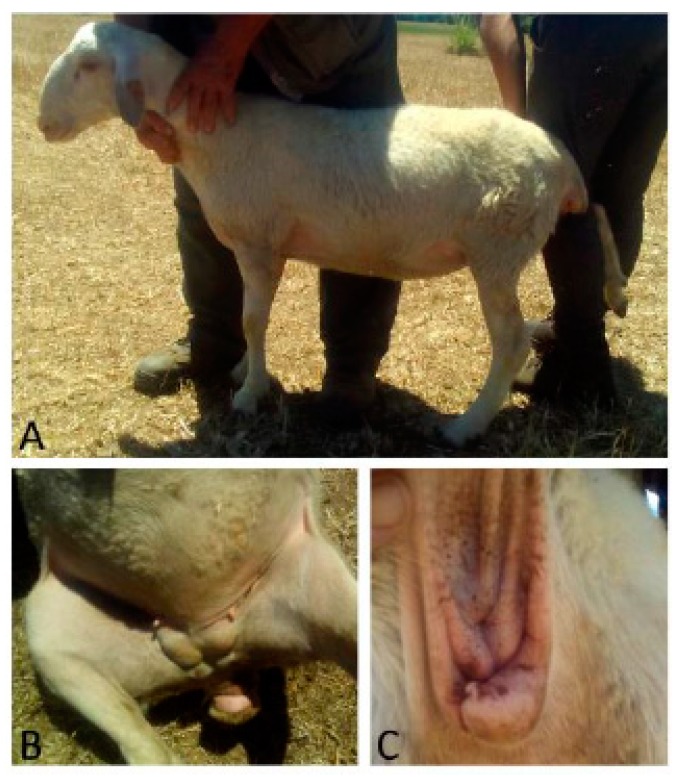
Case 2. (**A**) Montonino profile of the head; (**B**) two masses in the mammary region; (**C**) abnormal vulva.

Case 2 was an eight-month-old ewe of Comisana breed with male phenotype and montonino profile. The vulva opening was abnormal, and in the inguinal region, within the breast tissue, two testes-like structures were detectable (Figure 2). Sonographically, they showed a hyperechoic area well limited that enclosed a hepatized parenchyma with hypoechoic well-limited cavitations.

**Figure 3:**
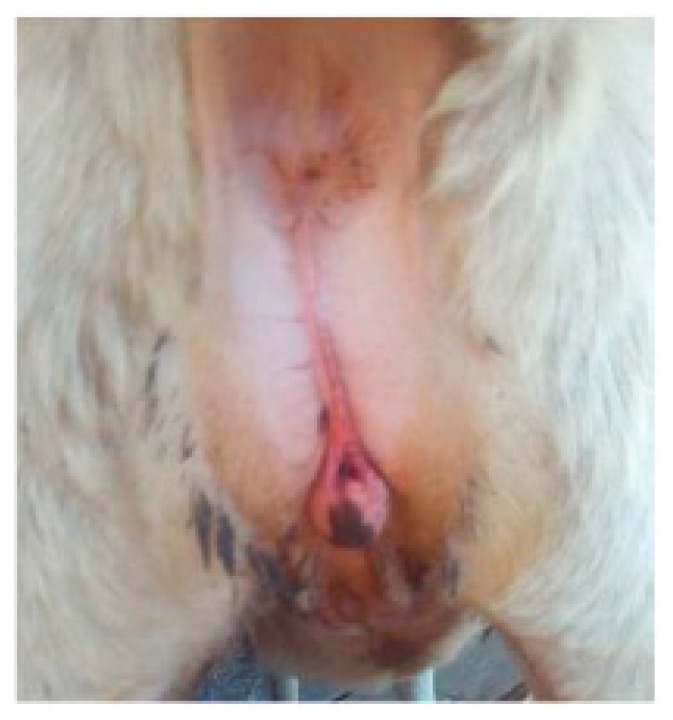
Case 3. Abnormal ano-vulva distance and enlarged clitoris.

Case 3 was a four-month-old ewe of Sarda breed with a longer ano-vulvar distance and an enlarged clitoris. Two testes-like structures were palpable at the inguinal level (Figure 3).

**Figure 4:**
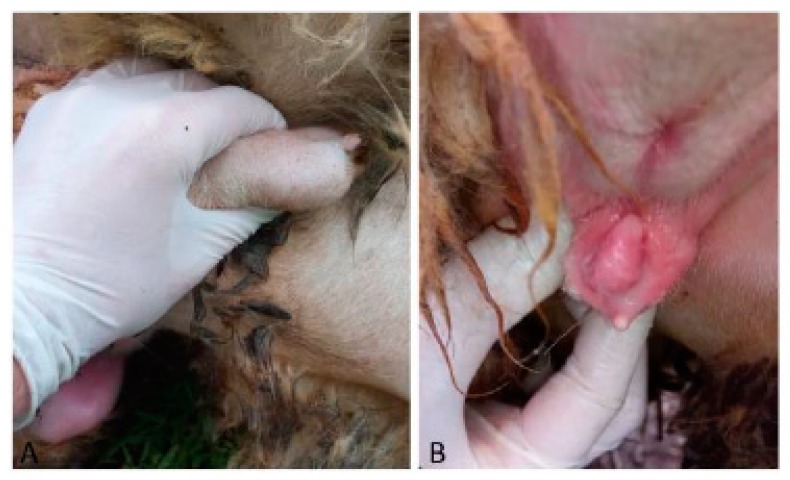
Case 4. (**A**) Isolation of the testis-like structure in the mammary region; (**B**) particular of the enlarged clitoris.

Case 4 was a 13-month-old half-breed ewe. The vulva was hypoplastic, and a penis-like structure protruded from the ventral commissure. In the mammary region, two testes-like structures were palpable (Figure 4).

### 3.2. Morphological Analyses

**Figure 5:**
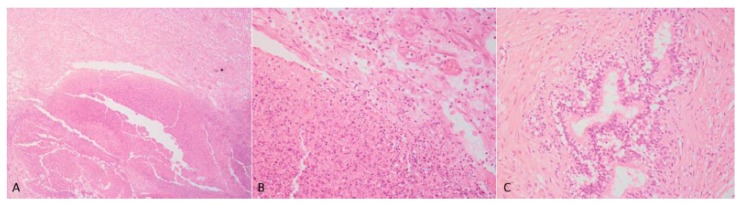
(**A**,**B**) Diffuse caseo-necrotic areas were admixed with degenerate neutrophils and surrounded by foamy macrophages. Normal parenchyma was almost totally replaced by necrosis and inflammation; (**C**) chronic epididymitis with inflammatory infiltrate consisting mostly of lymphocytes associated with interstitial fibrosis and epididymal epithelial mild hyperplasia. Haematoxylin and eosin staining.

**Figure 6:**
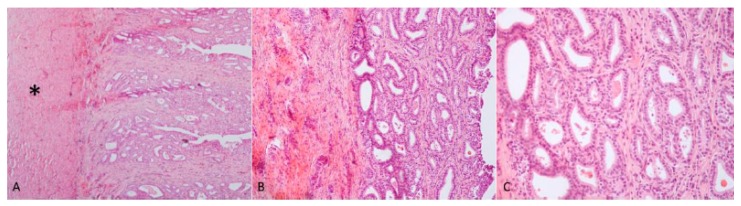
(**A**) Endometrial tissue characterized by an outer layer consisting of connective and hypo-trophic muscle tissue (asterisk) and an inner layer consisting of stratified pavement epithelium; (**B**,**C**) the epithelium was characterized by numerous, simple and slightly hyperplastic tubular uterine glands. Haematoxylin and eosin staining.

Case 1: The testes were grossly enlarged up to two or three times their normal size (25 × 20 cm). In the cut surface, there was a massive abscess and severe, diffuse, dry, grey-yellow necrosis resulting in the complete destruction of testicular parenchyma (Figure 1C). The epididymis was characterized by foci of diffuse thickening and fibrinous adhesions of the testicular vaginal tunics. Fibrotic tissue was also adherent to the tunic and scrotum. A tubular structure of small diameter with a cul-de-sac end and attached to the testis was observed. This structure was soft at sectioning, and the internal wall showed a diffusely pink surface and no fluid in the lumen (Figure 1D). Histological examination of the testis and epididymis revealed a diffuse area of caseo-necrosis, effacing 98% of the normal architecture and compressing, separating, surrounding and replacing the seminiferous tubules (Figure 5A). Necrotic debris was admixed with degenerate neutrophils, encircled by foamy macrophages (Figure 5B) and occasionally surrounded by granulation tissue and abundant collagen (fibrosis). In the remaining seminiferous tubules, there was diffuse germ cell atrophy with lack of spermatids. Epidydimal epithelia were surrounded by a mild, chronic, inflammatory infiltrate consisting mostly of lymphocytes and associated with severe fibrosis (Figure 5C). Microscopic examination of the tubular structure was interpreted as a uterine tube characterized by an outer layer of connective and hypo-trophic muscle tissue with few vessels and an inner layer compatible with endometrium (Figure 6A). The endometrial tissue was characterized by a stratified epithelium with numerous simple and slightly hyperplastic tubular uterine glands (Figure 6B,C). No ovarian tissue was macroscopically and microscopically visible.

### 3.3. Cytogenetic Analyses

The analysis of C-banded metaphases in Cases 1, 2 and 4 showed haematopoietic sex chromosome chimaeras 54; XX/XY with XY cell percentages of 8.7, 78.5 and 77.46, respectively.

**Figure 7:**
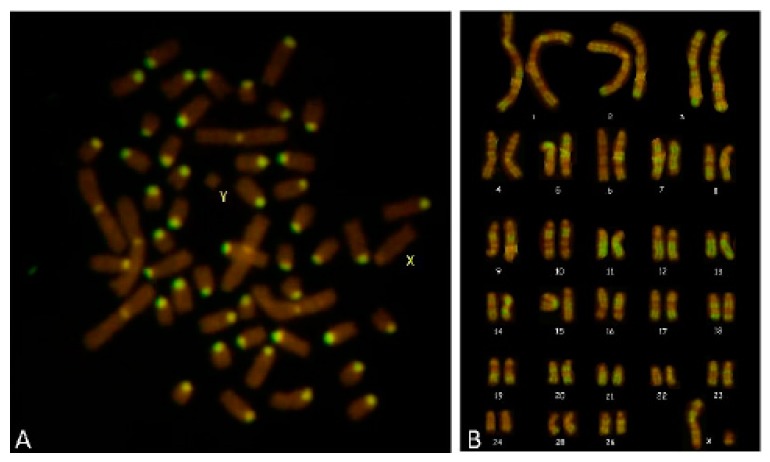
Case 3 ewe (2n = 54, XY). (**A**) C-banding pattern (CBA), sex chromosomes are labeled; (**B**) R-banding (RBA) karyotype.

Karyotyping of R-banded metaphases for all the cases showed a normal chromosome composition and the absence of any kind of aberration for all the cell clones. In Case 3, C-banding showed only 54, XY cells, and R-banding showed a normal karyotype (Figure 7). In regard to the heterosexual twins analysed, no one was affected by haematopoietic chimaerism according to the C-banding test.

### 3.4. Molecular Analyses

All the cases were *SRY*-positive and showed a double band in *AMELY/X* amplification corresponding to those on the Y and X chromosomes.

In regard to twins, the females of all the couples were negative for *SRY* gene amplification and showed only one band in *AMELY/X* amplification, corresponding to the fragment located on the X chromosome; the males showed the *SRY* gene and *AMELY/X*.

## 4. Discussion

Considering the number of DSD cases detected during this research, the actual incidence of this condition is underestimated by the breeders, at least in the first farm analysed, where the breeder declared one new DSD case/year, while in only 10 months, three DSD ewes were born. This is a confirmation of the fact that in sheep farming, DSD cases are often not detected, and only when farmers are alerted about this problem do they detect the cases.

In regard to the four analysed cases, they were all registered as females at birth and upon clinical examination, they showed a female phenotype with a high degree of masculinization. In particular, they showed an abnormal vulva with an enlarged clitoris similar to a penis and two obvious testes-like structures in the inguinal region, caudally to the mammary gland. In Case 1 also a blind-ending vagina was observed.

Cytogenetic analyses showed that Cases 1, 2 and 4 were haematopoietic cell chimaeras with different percentages of XY cells. Also, in this study, the percentage of XY cells in sheep with lymphocyte chimaeras is not related to the degree of development of the male reproductive tract, thus confirming the finding observed in previous studies on sheep [18], cattle [19,20], buffalos [21,22] and horses [23].

Unfortunately, no analyses were performed on fibroblastic cultures from these ewes. In addition, the available anamnestic data were not sufficient to establish whether they were born from multiple births with at least one male lamb, or if they have common ancestors. Thus, it is not possible to establish if they are true freemartin ewes or if they are the result of early zygotes or embryos fusions and if there is a genetic predisposition for vascular anastomosis in these cases. The analyses performed in the next birth season on 13 couples of heterosexual twins born in the same farm where Cases 1, 2 and 4 were detected, confirmed that freemartin syndrome is very rare and supported the hypothesis of early zygotes or embryos fusion.

Case 3 showed a female masculinized phenotype, *SRY*-positive, with a normal karyotype (Figure 7). In the literature, this is the second detected case of 54, XY *SRY*-positive DSD in sheep, and it showed a different phenotype. In fact, Ferrer et al. [15] reported the case of a ewe with normal external female genitalia, absence of masculinisation, and normal sexual behaviour, with a very short vulva opening (2.3 cm), a short blind-ending vagina (3.5 cm), and the total absence of any other male or female genital structure.

Another case of 54, XY sheep intersex was reported by Bruère et al. [13], but no SRY analyses were performed. Interestingly, this animal was born as a cotwin of a fertile ewe and was registered as a female at birth. Its body conformation was that of a ram, but it had a vulva without a clitoris and a short blind-ending vagina. No external gonads were palpable, and at laparotomy, two testes-like gonads were detected in ovarian position.

From a phenotypic point of view, both cases previously reported are different from Case 3, but the lack of complete anatomic and histopathological analyses prevents the possibility of correctly classifying this DSD, hypothesizing the molecular causes and stating if this is another expression of the same genetic condition of previous cases. XY *SRY*-positive DSDs have been reported for different animal species [24,25,26,27], and only in dogs has a molecular cause been identified [28]. Even so, the DNA sample of this case is of great importance, since it could be used in a future genomic analysis aimed to study this condition in sheep.

## 5. Conclusions

This paper reports the first results obtained from an initial screening of two sheep farms aimed to detect and study DSD in ovine species. Interestingly, as many as four cases of DSD were detected in only two farms, and of these, three chimaerisms were found in one and a case of XY *SRY*-positive intersex was found in the other. The finding of a greater number of DSD cases than expected by the breeder suggests that this problem in ovine species is underestimated by the sector operators and it would need more attention.

In regard to the cases of peripheral blood lymphocytes chimaerism, we were not able to ascertain its origin (from placental vascular anastomosis or early zygotes or embryos fusion). This data are intriguing both for deepening our knowledge about embryo development in mammals and for improving zootechnical management. In fact, an approximate 3% incidence of infertile ewe-lambs from male–female twin births may result in a significant number of freemartin ewe-lambs, potentially having important negative economic effects on flock profitability [5]. Thus, the hypothesis that the spreading of alleles responsible for multiple ovulations increases the risk of freemartinism in multiple births consolidates the need to sensitize farmers to the problem and to widen cytogenetic control in sheep farms. In addition to economic repercussions, the undiagnosed chimaerisms can hinder the scrapie eradication programs; in fact, the chimaeric females with ARQ/ARR genotype, because of the combination of the two cell populations, are not necessarily resistant to the disease [29].

The detection of a case of XY *SRY*-positive intersex ewe in a flock of only 50 animals suggests that gene mutations causing DSD in this species are present, although none have yet been identified, and it is therefore essential to extend these studies in ovine.

In sheep, like in other livestock species, reproduction and fertility are important aspects of management, and the early identification of individuals with DSDs undoubtedly entails economic advantages.

## Figures and Tables

**Table 1:** Primers sequences, annealing temperatures and product lengths of the analysed genes.

Gene	Primer Name	Primer Sequence	Annealing	Length
*SRY* * (Z30265)	*SRY*-F	CTG CTA TGT TCA GAG TAT TG	55	695bp
	*SRY*-R	TCA ATA TTG AAC ATA AGC GC		
*AMELX/Y* ** [17]	*AMELX/Y*-F	CAG CCA AAC CTC CCT CTG C	60	Y 240bp;
	*AMELX/Y*-R	CCC GCT TGG TCT TGT CTG TTG C		X 270bp

* Sex determining region Y; ** Amelogenin.
